# Defects of Vision or of Hearing in Relation to the Development of Children

**Published:** 1902-09

**Authors:** A. W. Stirling

**Affiliations:** Atlanta, Ga.


### DEFECTS OF VISION OR OF HEARING IN RELATION TO THE DEVELOPMENT OF CHILDREN.†

BY A. W. STIRLING, M.D.,

ATLANTA, GA.

I have pleasure in acceding to the request of our worthy chairman by saying a few words concerning the effects which may result from deficiency in the eyes and ears of children. During the time which I shall occupy, it will be impossibe to do more than outline this subject, but this I shall endeavor to do.

Let us first consider the eyes.

To begin immediately after birth, it may be well to remind those who have the care of children that the disease called ophthalmia of the new-born is acute and infectious, but almost always preventible by the thorough washing of the child's eyes after birth, and by dropping inside the lids for two or three days, a

* Prisoners and Paupers, page 131.

†Read before the Georgia Sociological Society, Atlanta, June, 1902.

solution of 10 per cent. of protargol, which pains little or not at all. How important it is to prevent the germ of this disease from obtaining a thorough lodgment in the eye will be at once apparent when the statement is credited that in Europe alone there are more than 300,000 persons now blind on account of its ravages, and that Credé in certain charitable institutions of Leipsic by the preventive treatment of the eyes of all the children born in them reduced the number of those affected from nearly 11 per cent. to about $\frac{1}{7}$ of 1 per cent. Of course in treating this affection the greatest care must be observed in order to prevent discharge from being conveyed by fingers, sponges, towels, etc., into the eyes of other persons.

Another disease, which is interesting from our present point of view, is that called trachoma or granular lids. It may have been observed that there has recently been a considerable sensation in New York over the prevalence of granular lids in the schools there, and that this complaint has been put upon the list of those to be notified to the Medical Officer of Health. Similar action has lately been advised in Atlanta, and will probably follow. I fear this proposal will carry consternation to many hearts in and around this city, because a large proportion of our local population is laboring under the delusion that they are possessed of granular lids. This belief is the effect of a loose use of words, and from this cause granular lids, like catarrh, have become a *bête noire* to very many people. The truth is that real granular lids are as uncommon at the present time here as they are common in some sections of cities like Berlin, Paris, London, and New York, especially in those sections in which the Hebrew and the Celtic races most do congregate. I have seen four cases among the white people since I came to Atlanta, some six and a half years ago ; none in negroes, in whom it is very rare. The fact that real granular lids are now so unusual here is no reason for the relaxation of vigilance in connection with them, because their number might increase, and because the disease is exceedingly troublesome and very dangerous to vision.

I shall say just a word about strabismus or squint. When the two eyes are not fixed exactly on the object looked at, but one deviates to either side, the object will appear to be double because two images of it are carried to the brain. The eyes should act as if they were one organ and not two. When they act as two separate organs they give rise to confusion in the brain. The brain objects to this, and soon refuses to take any notice of one of the images. The neglected eye is thus put out of use, and will remain so indefinitely unless suitable measures succeed in inducing the brain to take notice of both eyes at once. Even if the eyes are made straight by operation, the brain may still refuse to notice them simultaneously. As one eye is never so good as two, it is apparent that squints should be taken in hand at the earliest possible moment. Simple non-operative measures will then very frequently suffice for successful treatment.

Another very important disease is myopia or short sight. In speaking on this subject, I desire to dwell on the word disease in connection with it. It is usually due to a bulging of the back of the eye so that the rays of light do not focus upon the retina and cannot be made to do so except by spectacles or an operation. The bulging has a relationship to certain anatomical features in the orbit, but these are brought into play or accentuated by errors in the using of the eyes. Short sight usually begins after the child has begun to use the eyes for near objects, and after the elongation has once begun it is apt to increase till the age of seventeen or twenty, when it may cease to become worse; but it may even begin in adult life. The higher degrees of myopia especially are a danger to the integrity of the eye, because the stretching of its interior frequently causes tearing of its delicate membranes, with very serious results. To prevent short sight, children should not stoop over their work, or pore over their books; should not use their eyes till they are tired out, should use their eyes only on well illuminated objects of a size not likely to necessitate effort, the light not falling into the eyes, but coming by preference from over the left shoulder; their seats and desks should be properly fitted in school, and they should be specially careful during sickness. In some cases it is well even to relinquish near work altogether for a time.

Two other ocular conditions are often mistaken for short sight. One is astigmatism, and most eyes are more or less astigmatic.

This may be combined with short sight. It consists in an unequal curvature of the cornea, or clear front part of eye, so that the light does not all focus at one place and things appear more or less blurred. The unconscious effort to overcome this blurring by the use of the muscle of accommodation inside the eye is one of the most common causes of headache.

The only cure for this is eyeglasses

The other condition of the eye referred to is that which is common in the lower animals and in babies—an eye that is short from before backwards, the opposite of short sight and erroneously called long sight. This also may be combined with astigmatism. When the eye is too short—hypermetropia—the light can usually be focused by an effort of the muscle inside the eye, but this effort is tiring and also gives rise to headache. At a certain and quite early age the power of focusing diminishes, and vision becomes blurred. Glasses are the remedy for this defect also.

It is evident that a child with any one of these ocular defects is handicapped in proportion to its severity, and as a matter of fact children often lose a great deal of the possible benefit of their education from being so situated. Their lives are also not infrequently embittered in the same way, and they are set down as stupid or willful by teachers and companions, when in reality they do not see properly.

Deaf children are handicapped just in the same way; and, like defects of sight, defects of hearing can very frequently be prevented, and ought to be. The ears become diseased in children from two chief causes, viz.: Acute infectious diseases and an enlargement of a certain gland above the palate at the back of the nose. I believe, however, that in the inflammations of the ear due to infectious diseases it will yet be shown that very frequently it is through the medium of this gland that the inflammation spreads to the ear. The great hope of diminishing ear disease in children lies in treatment of these adenoids, as the enlarged glands have been called. It is remarkable how greatly adenoids will affect the health. When their effect is very marked they cause the child to *look* stupid—open mouth, heavy, expressionless face; they cause him also to *be* stupid, or at least to be less bright than he would otherwise be, because they interfere with the escape of some of the fluid from the brain which drain away in their situation; they interfere with sleep by preventing the free ingress and egress of air; and in the same way cause snoring and nightmare; they alter also the shape and development of the nose and palate; they may cause so-called bilious attacks from the swallowing of their discharge; and they, by a combination of circumstances, frequently greatly interfere with the general health, as the effect of their removal very often proves. The ventilating pipes from the ear to the throat, called the Eustachian tubes, open near them, and it is on account of this fact that earache, and suppurating ears are generally in children the result of adenoids, or possibly of enlarged tonsils alone, though these are apt to coexist.

The importance of disease of the ear as regards life itself it is well to bear in mind, because, as all life-assurance companies will testify, no one with perforation of the ear drum is safe from an attack of inflammation involving the bone behind the ear which frequently necessitates a prolonged operation in order to prevent an affection of the brain liable to end fatally.

For the full growth and development, mental and physical, of children, it is very important indeed that this matter of adenoids, with all that they entail, should be kept before parents and guardians, especially as their proper treatment produces results than which there are, on the whole, few, if any, more satisfactory in the whole domain of surgery.

